# Clinical characteristics of Japanese patients with epithelioid hemangioendothelioma: a multicenter retrospective study

**DOI:** 10.1186/s12885-018-4934-0

**Published:** 2018-10-19

**Authors:** Satoshi Shiba, Hiroshi Imaoka, Kazuhiko Shioji, Eiichiro Suzuki, Shigeru Horiguchi, Takeshi Terashima, Yasushi Kojima, Tatsuya Okuno, Yasutaka Sukawa, Kunihiko Tsuji, Kumiko Umemoto, Akinori Asagi, Akiko Todaka, Makoto Ueno, Masafumi Ikeda, Chigusa Morizane, Junji Furuse

**Affiliations:** 1 Department of Hepatobiliary and Pancreatic Oncology, National Cancer Center Hospital, Tokyo, Japan; 2 Department of Gastroenterology, Aichi Cancer Center Hospital, Nagoya, Aichi Japan; 3 Department of Internal Medicine, Niigata Cancer Center Hospital, Niigata, Japan; 4 Department of Gastroenterology, Graduate School of Medicine, Chiba University, Chiba, Japan; 5 Department of Gastroenterology and Hepatology, Okayama University Graduate School of Medicine, Dentistry, and Pharmaceutical Science, Okayama, Japan; 6 Department of Gastroenterology, Kanazawa University Hospital, Kanazawa, Japan; 7 Department of Gastroenterology, National Center for Global Health and Medicine, Tokyo, Japan; 8 Department of Medical Oncology, Kinki University, Faculty of Medicine, Osaka, Japan; 9 Division of Gastroenterology and Hepatology, Department of Internal Medicine, School of Medicine, Keio University, Tokyo, Japan; 10 Center of Gastroenterology, Teine Keijinkai Hospital, Sapporo, Japan; 11 Department of Hepatobiliary and Pancreatic Oncology, National Cancer Center Hospital East, Kashiwa, Japan; 12 Department of Gastroenterology, Shikoku Cancer Center, Matsuyama, Japan; 13 Division of Gastrointestinal Oncology, Shizuoka Cancer Center, Nagaizumi, Shizuoka Japan; 14 Department of Gastroenterology, Hepatobiliary and Pancreatic Medical Oncology Division, Kanagawa Cancer Center, Yokohama, Japan; 15 Department of Medical Oncology, Kyorin University Faculty of Medicine, Tokyo, Japan

**Keywords:** Epithelioid hemangioendothelioma, Prognosis, Tumor size, Ki-67 index, Sarcoma, Chemotherapy

## Abstract

**Background:**

Epithelioid hemangioendothelioma is an exceedingly rare sarcoma often occurring as an indolent angiocentric vascular tumor at various anatomic sites. Few reports have evaluated large case series of epithelioid hemangioendothelioma.

**Methods:**

We conducted a retrospective analysis of the clinical data of 42 consecutive patients with epithelioid hemangioendothelioma who were pathologically diagnosed between 1990 and 2014 at 13 Japanese tertiary hospitals. We analyzed their clinical characteristics, tumor features and prognostic factors.

**Results:**

The study included 22 men and 20 women, with a median age of 54 (range, 18–78) years. Pain was the most common symptom, occurring in 15 (68%) of the 22 symptomatic patients. The median maximum tumor diameter was 4.0 (range, 1.0–12.8) cm. The most commonly involved organs were the liver (81%), lungs (57%), and bones (12%). The overall survival rates were 79.5% at 1 year and 72.0% at 5 years. Substantially better survival was observed in asymptomatic patients than in symptomatic patients (*P* = 0.03), and better survival was also ovserved in patients with Ki-67 index ≤10% than in those with Ki-67 index > 10% (*P* = 0.04). By multivariate analysis, tumor size > 3.0 cm was associated with decreased survival (*P* = 0.049, hazard ratio 13.33).

**Conclusions:**

This study showed the clinical characteristics of Japanese patients with epithelioid hemangioendothelioma. Tumor size > 3.0 cm is an independent indicator of a poor prognosis in epithelioid hemangioendothelioma. The presence of symptoms at the time of diagnosis and high Ki-67 index implied poor survival.

## Background

Epithelioid hemangioendothelioma (EHE) is an exceedingly rare sarcoma (< 1 per 1 million population) [1] that often occurs as an indolent angiocentric vascular tumor at various anatomic sites [2, 3]. It originates from endothelial-like cell with a clinical behavior intermediate between hemangioma and angiosarcoma [2]. Although World Health Organization has recommended that EHE be grouped with angiosarcomas [4], it has an unpredictable clinical behavior ranging from indolent to aggressively malignant [5]. Previous studies showed that the 5-year disease-specific survival for EHE was 81% in contrast to approximately 50% mortality rate at 1 year for soft-tissue angiosarcomas [6, 7]. The lung, liver, bone, and soft tissue are the most common involved sites [3], and this has been supported by similar data in other studies [2, 8]. The characteristics of the tumor are basically similar in the various organs while the clinical presentation and disease-related signs and symptoms differ. Most of the the lesions are peripheral with low-attenuation pattern on unenhanced computed tomography scans, some tumor nodules can be widespread with extensive confluent masses and display marginal enhancement on contrast-enhanced scans. The differential diagnosis for EHE includes vascular malignancies such as epithelioid angiosarcoma, and other epithelioid tumors. Immunohistochemistry can also be helpful in the diagnosis, and the combination of Fli-1 and CD31 has been suggested to identify EHE. Recently, a diagnosis of EHE was reinforced by the finding of two novel disease-defining gene fusions, namely, *WWTR1(TAZ)-CAMTA1* and *YAP1-TFE3*, that were detected in nearly 90 and 10% of EHEs, respectively [9–11]. It has been also reported that prognosis of EHEs is dissimilar according to the involved site, such as lung, liver, and soft tissue [3, 12]. A risk stratification method has been proposed to identify lesions at high risk for tumor progression, with the idea that they can be targeted for more aggressive therapy, such as curative resection and transplantation [13]. However, information about epidemiology, biology, and clinical behavior of this disease is lacking, and little is known of its prognosis. Therefore, we hypothesized the clinical behavior of EHEs was affected by patient demographics and tumor characteristics and then investigated the clinical information on EHEs in several Japanese centers from 1990 to 2014. Herein, we report the features of the patients with EHEs.

## Methods

Information about the patients with EHE who were pathologically diagnosed between 1990 and 2014 was obtained from 13 Japanese tertiary hospitals which belong to the working group associated with the hepatobiliary and pancreatic oncology group of the Japan Clinical Oncology Group. The clinical data of the consecutive EHE patients including patients’ demographic, tumor feature, treatments, and survival were retrospectively analyzed because the incidence rate of EHE was extremely low. Case report forms were sent to the hospitals to retrieve the needed data. Statistical analyses with cross-tables, the Fisher’s exact test, and Cox regression analysis were performed using SPSS ver. 19 (IBM, Chicago, IL, USA). The survival period was calculated using Kaplan-Meier estimates from the date of initial diagnosis to the date of death or last clinical follow-up. Furthermore, age (< 55, ≥ 55) [1], sex (female, male), tumor size (≤ 3.0 cm, > 3.0 cm) [6], Ki-67 labeling index (≤ 10%, > 10%: Using a monoclonal antibody, Ki-67, which reacts with a nuclear antigen in proliferating cells, the percentage of Ki-67 positive cells was assessed.) [7], symptoms (absence, presence), and organ involvement (single, multiple) [1] were classified as categorical values referring previous reports and evaluated via univariate and multivariate analyses. Institutional ethical approval was obtained for this project before starting data collection and written informed consent was waived owing to the retrospective nature of the study.

## Results

### Demographics

A total of 42 patients comprising 22 men and 20 women with a median age of 54 (range, 18 to 78) years were included in the study. The patient characteristics are summarized in Table 1. All the patients had confirmed pathological diagnosis of EHE. Twenty-two patients (52%) were symptomatic at diagnosis, with pain being the most common presenting symptom (15/22, 68%). The details of the presenting symptoms at diagnosis are summarized in Table 2.

**Table 1:** Baseline characteristics of the patients

	No. (%) of patients (*n* = 42)	
Age, median (range)	54 (18–78)	
Sex		
Male	22	(52)
Female	20	(48)
Smoking history^a^		
Present	17	(40)
Absent	23	(55)
Unknown	2	(5)
Symptoms at diagnosis		
Present	22	(52)

^a^Seventeen patients with smoking history included 14 males (64%) and 3 females (15%). In comparison with a large-scale population study in Japan concerning total cancer incidence according to smoking status, 84% of males and 9% of females at the diagnosis of cancer had smoking history [24]

**Table 2:** Symptoms at diagnosis

Symptoms (*n* = 42)	No.	(%)
Abdominal pain	6	(18)
Back pain	4	(12)
Palpable tumor	3	(9)
Weight loss	3	(9)
Fatigue	3	(9)
Cough	3	(9)
Epigastric pain	2	(6)
Chest pain	2	(6)
Pyrexia	2	(6)
Neck pain	1	(3)
Vocal cord paralysis	1	(3)
Bronchial pneumonia	1	(3)
Respiratory discomfort	1	(3)
Bloody sputum	1	(3)

Twenty-two patients of all the patients (*n* = 42) who had symptoms at the time of diagnosis are included on this table, and percentages for each sign and symptom are given as the fraction of 42 patients. Patients often reported more than one symptom

### Tumor features

EHEs were identified in various sites and organs. Twenty-five patients (60%) presented with metastatic disease, that is, multiple organ involvement, at diagnosis. The most commonly involved organs were the liver (*n* = 34, 81%), lung (*n* = 24, 57%), and bone (*n* = 5, 12%), which were identified to have multifocal lesions. The other involved organs are summarized in Table 3. Nineteen patients (45%) had both liver and lung involvement. Meanwhile, 17 patients (40%) had single-organ involvement, the most common organ of which was the liver (*n* = 11, 65%), followed by the lung (*n* = 4, 23%), bone (*n* = 1, 6%), and skin (*n* = 1, 6%). The median maximum tumor diameter was 4.0 (range, 1.0 to 12.8) cm.

**Table 3:** Tumor characteristics of the patients with EHEs

Characteristics (*n* = 42)	No.	(%)
Maximum tumor diameter (cm)		
Median, range	4.0 (1.0–12.8)	
Median (range) number of organs involved	2 (1–5)	
Organ involvement		
Single	17	(40)
Multiple (two or more)	25	(60)
Organ involved^a^		
Liver	34	(81)
Lung	24	(57)
Bone	5	(12)
Skin/Subcutaneous tissue	3	(7)
Lymph node	2	(5)
Spleen	2	(5)
Heart/Pericardium	2	(5)
Brain	1	(2)
Parotid gland	1	(2)
Stomach	1	(2)
Peritoneum	1	(2)

^a^ Patients with multiple metastases are overlapping according to the number of the involved organs

### Treatments

As regards initial treatments, 11 patients (26%) underwent curative resection for their EHEs, while 31 patients (74%) received non-curative treatments. The most first-line non-curative treatment was systemic chemotherapy (*n* = 10, 24%). For seventeen patients (40%), observation/watchful waiting was performed for the observational period. More than 50% of the patients had metastatic disease in this study, and they underwent various treatments (Table 4). In patients with single-organ involvement, initial treatments were curative resection (*n* = 8), systemic chemotherapy (*n* = 2), transcatheter arterial chemoembolization (*n* = 1) and watchful waiting (*n* = 6). In patients with multi-organ involvement, initial treatments were systemic chemotherapy (*n* = 8), curative resection (*n* = 3), dubulking surgery (*n* = 1), radiofrequency ablation (*n* = 1), radiation (*n* = 1) and watchful waiting (*n* = 11).

**Table 4:** Initial treatments for the patients with EHEs

Initial treatments (*n* = 42)	No.	(%)
Observation/watchful waiting	17	(40)
Curative resection	11	(26)
Debulking surgery	1	(2)
Radiofrequency ablation	1	(2)
Radiation	1	(2)
Transcatheter arterial chemoembolization	1	(2)
Systemic chemotherapy	10	(24)

### Systemic chemotherapy

Ten patients (24%) were treated with systemic chemotherapy as initial therapy. The most commonly used regimen were paclitaxel (*n* = 3, 7%) and the combination of carboplatin, paclitaxel, and bevacizumab (*n* = 3, 7%). The anti-angiogenic drugs including pazopanib (*n* = 2), thalidomide (*n* = 1), and bevacizumab (*n* = 1) were also used, and no patients who received these drugs achieved partial response (PR). Among them, 4 patients received 2nd-line chemotherapy, and 3 patients received 3rd-line and more. Miscellaneous regimens were used for the patients with unresectable EHEs (Table 5).

**Table 5:** Systemic chemotherapy for the patients with EHEs

Systemic chemotherapy regimen	No.	(%)	Individual response
1st-line treatment	10	(100)	
Carboplatin + paclitaxel + bevacizumab	3		PR, SD, PD
Paclitaxel	2		SD, SD
Pazopanib	2		SD, SD
Bevacizumab	1		SD
Streptozocin	1		NE
Cisplatin + epirubicin + bevacizumab	1		PD
2nd-line treatment	4	(40)	
Carboplatin + pemetrexed + bevacizumab	1		PR
Thalidomide	1		PD
Irinotecan	1		SD
Interleukin-2	1		SD
3rd-line treatment	3	(30)	
Adriamycin	1		SD
Paclitaxel	1		SD
Pemetrexed + bevacizumab	1		SD
4th-line treatment	1	(10)	
Investigational drug	1		SD
5th-line treatment	1	(10)	
Investigational drug	1		SD

*PR* partial response, *SD* stable disease, *PD* progressive disease, *NE* not evaluated

A patient who received the combination regimen of carboplatin, paclitaxel, plus bevacizumab as 1st-line and carboplatin, pemetrexed, plus bevacizumab as 2nd-line had PR as best response to both the combination regimens.

### Survival

**Fig. 1:**
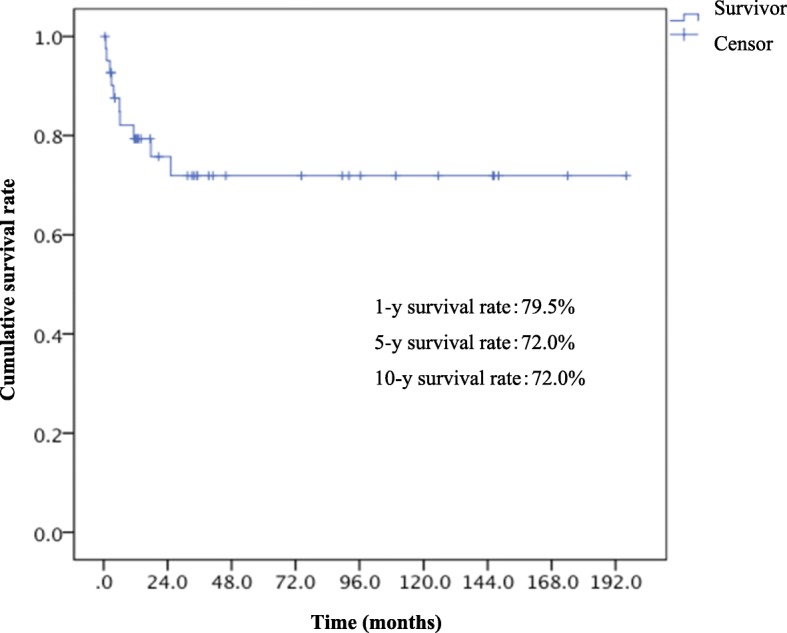
Overall survival of the 42 patients with EHEs

**Fig. 2:**
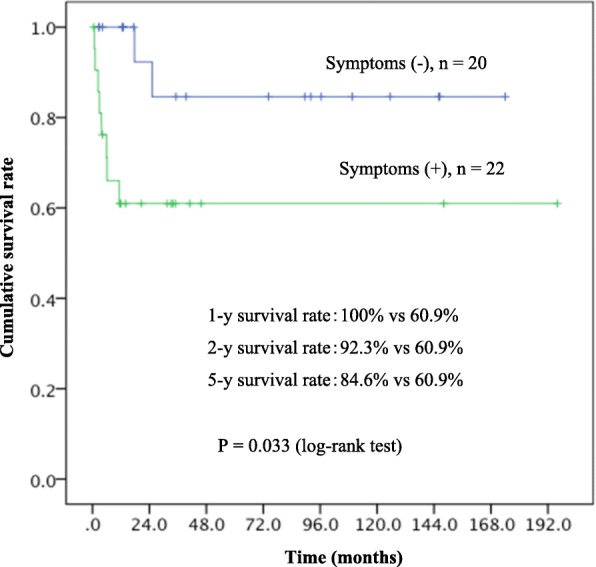
Survival curve according to the presence of symptoms at the time of diagnosis

**Fig. 3:**
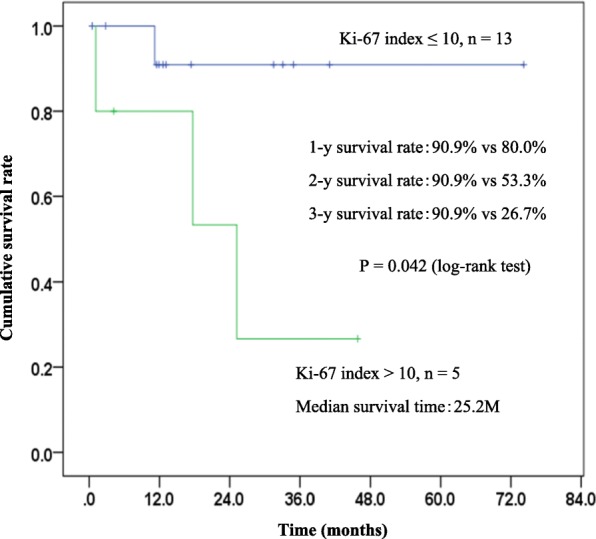
Survival curve according to the Ki-67 labeling index

The Kaplan-Meier plot of overall survival for all the patients (*n* = 42) is shown in Fig. 1. The 1-, 5-, and 10-year survival rates were 79.5, 72.0, and 72.0%, respectively. The median survival time of all the patients was not reached. The symptomatic patients at EHE diagnosis showed significantly poorer prognosis than asymptomatic patients (*P* = 0.03, log-rank test) (Fig. 2). The patients with Ki-67 index > 10% exhibited significantly poorer prognosis than those with Ki-67 index ≤10% (*P* = 0.04, log-rank test) (Fig. 3). Multivariate analysis showed that tumor diameter > 3 cm was significantly associated with risk of death (hazard ratio [HR]: 13.33; 95% confidence interval [CI]: 1.01–175.52, *P* = 0.049) (Table 6).

**Table 6:** Cox regression model for overall survival in all the patients with EHEs (*n* = 42)

	Univariate analysis			Multivariate analysis		
	HR	95% CI	*P* value	HR	95% CI	*P* value
Age (years)						
< 55	Ref			Ref		
≥ 55	1.249	0.361–4.316	0.725	5.804	0.860–39.176	0.071
Sex						
Female	Ref			Ref		
Male	0.919	0.266–3.175	0.893	1.602	0.336–7.638	0.555
Symptoms						
Absence	Ref			Ref		
Presence	4.643	0.983–21.928	0.053	3.973	0.774–20.384	0.098
Tumor diameter (cm)						
≤ 3.0	Ref			Ref		
> 3.0	5.842	0.739–46.182	0.094	13.327	1.012–175.520	0.049
Organ involvement						
Single	Ref			Ref		
Multiple	0.721	0.209–2.493	0.606	0.311	0.065–1.479	0.142
Ki-67 labeling index^a^						
≤ 10	Ref					
> 10	7.526	0.770–73.558	0.083			

*HR* hazard ratio, *CI* confidence interval, *Ref* reference

^a^ The Ki-67 labeling index was not evaluated by multivariate analysis because its value in 24 of the 42 patients was unknown

## Discussion

In the present study, we investigated the clinical features and treatments of 42 patients with EHEs at 13 Japanese tertiary hospitals. Few studies have conducted a multicenter evaluation of EHE, which is an exceedingly rare and unique sarcoma. To our knowledge, this is one of the largest published multi-institutional cohorts of patients with EHEs ever reported. Although our study is limited by its retrospective nature, novel data on EHE have been obtained. The clinical diagnosis and treatments for patients with such malignancy remains a challenge for clinicians. The patient characteristics, such as age, female-to-male ratio, involved organs, tumor features, symptoms at diagnosis, and prognosis, in Japanese patients with EHEs are similar to those in previous reports [1, 6]. The survival rates from our study are generally same as those reported in Western countries [1, 6, 4].

Twenty-two patients (52%) presented with various symptoms derived from the site of EHEs. The symptoms were not only local, such as pain, cough, and palpable mass, but also systemic, such as weight loss, fatigue, and pyrexia. These systematic symptoms seem to be induced by some cytokines released from the EHEs [15]. Interestingly, the symptoms noted in the present study tended to be similar to those previously reported, with pain being the most common symptom. However, the proportion of symptomatic patients was higher in Western countries (72%) [1] than that in Japan (52%, Table 1).

Various treatments were performed for the patients with EHE in this study. Sugical resection can be curative for EHE and it was performed in 11 patients (26%) as initial treatment. Debulking surgery (*n* = 1), radiofrequency ablation (*n* = 1), radiation (*n* = 1), transcatheter arterial chemoembolization (*n* = 1), and observation/watchful waiting (*n* = 17) were also performed, which are not commonly done for EHE in Western countries (Table 4). Additionally, wathchful waiting is sometimes a reasonable strategy for patients with EHE because it was actually done in 17 patients in this study. Concerning systemic chemotherapy, paclitaxel was frequently used in both Japan and Western counties. Clinical trial of weekly paclitaxel for patients with angiosarcoma: the ANGIOTAX study [16] reported a median time to progression of 4 months for metastatic angiosarcoma, a disease belonging to the same group of vascular sarcoma, and this might be a basis for deciding on the treatment regimen for patients with EHEs. Combination regimens are often used in Japan (Table 5), while monotherapy is mainly used in Western countries [14]. Anti-angiogenic drugs, such as bevacizumab, pazopanib, sorafenib, sunitinib, and axitinib and the mammalian target of rapamycin (mTOR) inhibitor, such as sirolimus are used in Western countries [14, 17–20]. Concerning the efficacy of systemic chemotherapy, our findings showed that the combination regimens of carboplatin, paclitaxel plus bevacizumab or carboplatin, and pemetrexed plus bevacizumab achieved PR as evaluated using the Response Evaluation Criteria In Solid Tumors version 1.1 (Table 5). In contrast to the findings of the present study, patients treated with systemic chemotherapy using single regimens such as interferon, celecoxib, bevacizumab, sorafenib, pazopanib, and thalidomide in previous studies were confirmed to have achieved PR [14, 17–19, 21]. The combination of carboplatin and bevacizumab is interesting because the efficacy of those combination regimen for EHE have never been reported, and these regimens should be explored in clinical trials. Concurrently, role of systemic therapy and its efficacy for advanced EHEs need further investigation. Novel gene fusions with oncogene properties, namely, *WWTR1(TAZ)-CAMTA1* and *YAP1-TFE3*, are expected to be directly used as treatment in the future because *TAZ* and *YAP1* play major roles in the Hippo pathway, which regulates tissue homeostasis, organ size, cell regeneration, and tumorigenesis [22, 23].

Most EHEs are considered to be indolent; however, our data and previous reports have shown 20–60% of tumors metastasize, and approximately 15% of patients die of EHE [8, 14, 15]. Deyrup et al. reported that a combination of tumor size and mitotic activity has been useful to stratify tumors into low- and high-risk groups, that is, patients with tumors > 3 cm in diameter and > 3 mitoses per 50 HPF have a lower 5-year survival of 59% than the 100% survival rate in patients with tumors that lacked both features [6]. The result of multivariate analysis in the present study demonstrated that tumor diameter > 3.0 cm was associated with poor outcome (Table 6), and that patients with Ki-67 index > 10% suggested worse survival than those with Ki-67 index ≤10% (Fig. 3). As mentioned by the previous report [7], one of factors that corresponded with poor prognosis was high Ki-67 values (≥ 10%) in angiosarcoma which mimics EHE. This result implied that the Ki-67 index could be used to classify EHE into low- and high-risk groups. Moreover, the role of the Ki-67 index needs to be explored further because its value in 24 of the 42 patients in the present study was unknown. Our data support past reports proposing risk classification of EHE. The findings of the current study suggest that the presence of symptoms, most of which are caused by large tumors and may reflect performance status, is related to poor outcome (Fig. 2).

## Conclusions

This multi-institutional retrospective analysis of 42 patients with EHEs demonstrated the clinical features and treatments of the disease. Tumor size > 3.0 cm is identified as an independent poor prognostic factor in EHEs. The presence of symptoms at diagnosis and Ki-67 index > 10% might be correlated to poor outcome. Additional clinical and molecular tumor data are needed to define possible subgroups in order to individualize future treatment of patients with EHEs.

## Abbreviations

CAMTA: Calmodulin-binding transcription activator
CI: Confidence interval
EHE: Epithelioid hemangioendothelioma
Fli: Friend leukemia integration
HR: Hazard ratio
mTOR: Mammalian target of rapamycin
PR: Partial response
TAZ: Transcriptional co-activator with PDZ-binding motif
TFE: Transcription factor E
WWTR: WW domain containing transcription regulator
YAP: Yes-associated protein
